# DiTA: helping you search for evidence of diagnostic test accuracy in physical therapy

**DOI:** 10.1016/j.bjpt.2024.101082

**Published:** 2024-05-20

**Authors:** Mark A. Kaizik, Mark J. Hancock, Robert D. Herbert

**Affiliations:** a School of Biomedical Sciences, Faculty of Medicine and Health, University of New South Wales, Sydney, Australia; b Faculty of Medicine and Health Sciences, Macquarie University, Sydney, Australia; c Neuroscience Research Australia (NeuRA), Sydney, Australia

**Keywords:** Accuracy, Bibliographic database, Diagnosis, Physical therapy

## Abstract

**Background:**

Physical therapists use diagnostic tests in a variety of settings. Choosing the best diagnostic test to apply in a particular situation can be difficult. The choice of diagnostic test should be informed, at least in part, by evidence of test accuracy. Finding evidence of diagnostic test accuracy has, until recently, been challenging. Ideally, there would exist a database that comprehensively indexes evidence on diagnostic tests relevant to physical therapy practice, is free to access, and is easy to use.

**Objective:**

This Masterclass will describe the DiTA (*Di*agnostic *T*est *A*ccuracy) database (dita.org.au) including its development and search interface, and provide advice on how to search and retrieve records.

**Discussion:**

DiTA indexes more than 2400 primary studies and systematic reviews of diagnostic test accuracy relevant to physical therapy practice. Users can search DiTA using text fields and dropdown lists to find evidence of diagnostic test accuracy. The database is freely accessible on the internet. Since its launch, DiTA has been accessed from almost every country in the world, the largest number of searches having been conducted from Brazil.

## Highlights

• The DiTA database, launched in 2019, is freely accessible at dita.org.au.
• DiTA indexes physical therapy relevant diagnostic test accuracy research.
• There are more than 2400 primary studies and systematic reviews indexed on DiTA.
• This Masterclass provides tips on how to best search this database.

## Introduction

Diagnostic tests are used to assess the presence or absence of specific pathologies.^1^ It is helpful to distinguish diagnostic tests from other types of (non-diagnostic) tests used in clinical practice. Non-diagnostic tests are used for purposes such as assessing function, assessing impairment or monitoring progress (e.g., range of motion tests, muscle strength tests); or predicting future symptoms, conditions, or response to treatment (e.g., the STarT Back Tool for low back pain^2^). This Masterclass paper focuses on diagnostic tests. It describes the DiTA database and explains how DiTA can help physical therapists find evidence of diagnostic test accuracy.

Diagnostic tests are used in most subdisciplines of physical therapy.^3^ An example from musculoskeletal physical therapy is the use of Lachman's test for rupture of the anterior cruciate ligament of the knee^4^; an example from cardiorespiratory physical therapy is the PUMA COPD diagnostic questionnaire^5^; and an example from women's health physical therapy is the pad test for urinary incontinence.^6^

Clinicians conduct diagnostic tests in the expectation that the findings of the diagnostic test will increase certainty about whether a particular pathology is present or absent.^7^ A physical therapist often has multiple choices when selecting a diagnostic test to apply. However, the process of choosing between tests can be bewildering and it is often not clear which test is the best to choose.^8^ An example arises when a physical therapist assesses for the presence or absence of sacroiliac joint pathology in patients with low back pain.^9^ The physical therapist could consider using the distraction test, the compression test, the thigh thrust test^10^, ^11^, ^12^; Gaenslen's test^11^^,^^12^; the sacral thrust test^13^; the patient's report of pain over the area of the sacroiliac joint^14^^,^^15^; and various composites of these and other tests.^11^^,^^13^^,^^14^^,^^16^ A clinician's choice of a diagnostic test to apply can be influenced by many factors such as pattern recognition and heuristics,^17^ the patient's preference for a particular test,^18^ availability or ease of access to the test,^19^ and fear of litigation.^20^ Ideally the choice of test should be influenced by evidence of diagnostic test accuracy.^21^

### Evidence of diagnostic test accuracy

Evidence of diagnostic test accuracy can be found in reports of primary studies of diagnostic test accuracy and systematic reviews of primary studies of diagnostic test accuracy. In a typical primary study, both an index test and a reference standard are applied to subjects who are suspected of having a particular pathology and these test results are compared. The index test is the diagnostic test whose accuracy is being evaluated. The ideal reference standard (sometimes called a “gold standard”) is a diagnostic test that perfectly classifies the subject as either having or not having the pathology. In practice, it is rare to find perfect or near-perfect reference standards, so the reference standard used is generally the best available diagnostic test for the pathology being assessed.^22^ Researchers conduct studies to determine the accuracy of index tests because, while index tests are usually expected to be less accurate than the reference test, they may be preferred over reference tests, in some clinical contexts, because they are less expensive, more accessible, easier to conduct, less invasive, safer, or less painful than the reference test. For example, Lachman's test has been used as an index test for the diagnosis of anterior cruciate ligament rupture, rather than the best reference test of arthroscopy. However, if an index test is to be used in clinical practice it must be sufficiently accurate to aid, rather than confuse, diagnosis.

Primary studies of diagnostic test accuracy compare the findings of the index test to the reference standard. The similarity of those findings provides a measure of the diagnostic accuracy of the index test. Accuracy can be quantified and reported in a variety of ways. Most commonly accuracy is reported in terms of sensitivity, specificity, positive predictive value, negative predictive value, positive likelihood ratio, and negative likelihood ratio.^23^ Other accuracy measurements include Youden's index,^24^ diagnostic odds ratios,^25^ receiver operating characteristic (ROC) curves and the area under ROC (AUROC) curves,^26^ and a measure more simply known as accuracy, which is the ratio of the combined true positive and true negative cases to the total number of cases evaluated.^27^

When more than one primary study has investigated the accuracy of a particular index test, researchers may conduct a review of those studies. Ideally the review uses a systematic review methodology. Systematic reviews of primary studies of diagnostic test accuracy use similar methods to systematic reviews of primary studies of intervention. Ideally, the review protocol is pre-specified, a research question is clearly posed, a comprehensive search for primary studies is conducted to retrieve and select appropriate studies, and the quality of the studies is assessed. Results are then summarised and conclusions are drawn.^28^, ^29^, ^30^ In systematic reviews of diagnostic test accuracy studies, data from multiple primary studies are pooled with the aim of providing more precise estimates of test accuracy such as sensitivity or specificity.^7^^,^^31^^,^^32^

The alternative to a systematic review is a narrative review. Narrative reviews also summarise the evidence provided by primary research studies but typically fail to specify the sources and search strategy of included studies, do not explicitly nominate the criteria used to select studies for inclusion in the review, and summarise data qualitatively rather than quantitatively.^33^ Consequently, it is thought by many methodologists that narrative reviews are more likely to generate biased conclusions than systematic reviews. For that reason, systematic reviews are generally preferred to narrative reviews.

### The DiTA database

Ideally, there would exist a database that provides a comprehensive listing of physical therapy-related primary diagnostic test accuracy studies and related systematic reviews, is freely accessible to all, and is easy to use. In the past, it has been challenging for physical therapists to easily access evidence of diagnostic test accuracy. Thus, while PubMed is a comprehensive and freely available database of biomedical literature, it is not easy to find evidence on diagnostic test accuracy on PubMed because it indexes over 36 million records of which only a very small proportion are studies of diagnostic test accuracy.^34^ The Cochrane Collaboration publishes a freely available register of systematic reviews, some of which are diagnostic test accuracy reviews, but it contains few reviews of diagnostic test accuracy relevant to physical therapy.^35^ The Physiotherapy Evidence Database (PEDro; pedro.org.au) is a database that indexes primary studies and systematic reviews relevant to physical therapy; however, PEDro indexes evidence of the effects of interventions and not on the accuracy of diagnostic tests.^36^

In response to the need for a comprehensive, free, easy to use database of studies of diagnostic test accuracy relevant to physical therapy, the DiTA database^37^ (*Di*agnostic *T*est *A*ccuracy database; dita.org.au) was launched in 2019. It was developed over a period of four and a half years by a team that was largely made up of Steering Committee members and volunteers of PEDro. DiTA's architecture, website, search interface, search fields, and layout are similar to the PEDro database, so regular users of PEDro will find DiTA easy to use. Following a large scale initial search of the literature to seed the database,^3^ DiTA's records have been updated on a monthly basis since July 2019 by staff with specific expertise in searching the physical therapy research literature to identify relevant studies using DiTA's published inclusion criteria.^38^ For a paper to be indexed on DiTA, it must evaluate a diagnostic test procedure relevant to physical therapy. The diagnostic test procedure must be able to be performed by a physical therapist and not just produce results that a physical therapist would use (so, for example, a study evaluating the accuracy of a radiograph would not be included). There are no language restrictions for the literature indexed on DiTA.

**Fig. 1:**
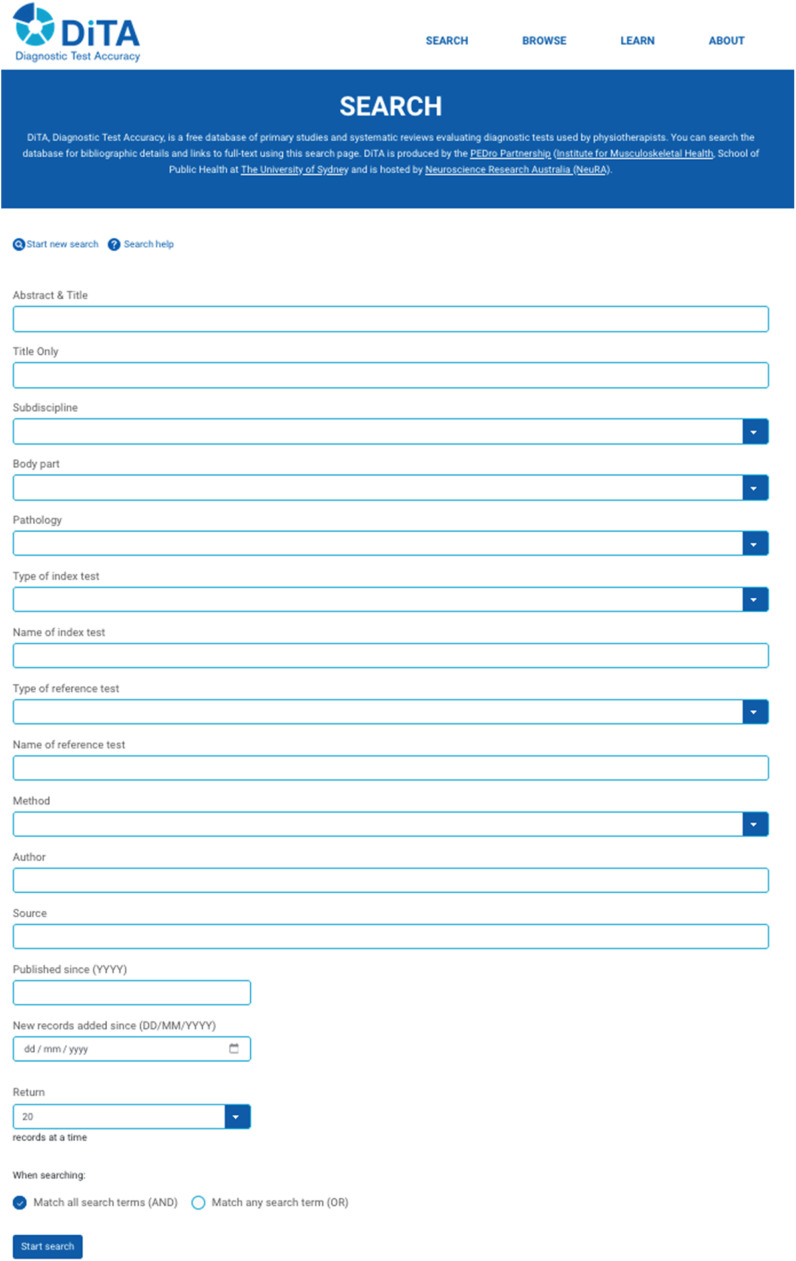
DiTA search page.

To search the database, users must first navigate to the freely available DiTA search page (search.dita.org.au; Fig. 1). On this page, the user can nominate a search query by entering text into search fields or selecting dropdown list items. Fields include “Abstract & Title” to search for search terms in the abstract or title of indexed papers; “Subdiscipline” to search for papers related to a specific subdiscipline in physical therapy such as ‘neurology’ or ‘cardiothoracics’; “Body part” to search for papers related to a specific part of the body such as ‘head or neck’, or ‘perineum or genito-urinary system’; and “Pathology” to search for papers related to broad categories of pathology such as ‘muscular’ or ‘articular’. Characteristics of the index test and reference standard can also be searched. For example, “Type of index test” allows the user to search for a specific type of diagnostic test being evaluated in a study such as a ‘questionnaire’, a ‘physical examination’ procedure, a ‘health technology’ like ultrasound or spirometry, or an index test that is a mixture of any of these types (‘mixed’). Users can also specify what type (“Method”) of indexed paper they want their search to query (‘primary study’ or ‘systematic review’), as well as search for a specific author name (“Author”) or a journal name (“Source”) that may help them retrieve publications of interest.

Users have an option at the bottom of the search page to combine all their search field queries using the Boolean operators of ‘AND’ or ‘OR’. ‘Match all search terms (AND)’ is the default option. If more than one word is entered into a text field, the words in that specific field are automatically combined using the Boolean operator ‘AND’ even if the user has also selected to combine all their search field queries at the bottom of the search page using ‘OR’. When DiTA automatically combines search terms in a specific field using ‘AND’ this way, each word must be present in that queried field (e.g. the “Title” field) of a record for it to be retrieved although the words don't need to be adjacent to each other. Alternatively, inclusion of more than one search term within inverted commas (“ ”) will tell DiTA to only look for records containing all of those terms in that order. Terms can be entered into a text field using wildcards (e.g. ‘*’) that search for variations of words in that field. Searching for ‘*edema’ in the “Title” field will retrieve records that have titles containing ‘edema’, ‘oedema’, ‘lymphedema’ or ‘lymphoedema’.

If records exist on the database that match the search query, they will be listed in table format showing the “Title” and the “Method” of research used for each displayed record (‘primary study’ or ‘systematic review’). Specific records can be selected from this list to view in more detail at a later time. Alternatively, more details of a record can be viewed directly from the table by clicking on the record's title. The extra details include the authors, source, and abstract of the paper (if copyright release has been granted). DiTA does not give direct access to whole papers. However, DiTA does provide links to online platforms that may (or may not) provide the full electronic manuscript. Some papers are available in full text for free (Table 1).

**Table 1:** Top 4 tips on how to search DiTA for evidence of diagnostic test accuracy.

1	Limit the initial search to using just one or two search fields. (Use the “Abstract & Title” field if only using one field to search.)
2	When using the “Abstract & Title” field, just use two or three simple search terms separated with spaces and not with Boolean operators. Boolean operator functions are best controlled using the options at the bottom of the search page.
3	Check all spelling is correct, particularly the use of apostrophes for specific index test names (e.g. search for “Lachman's” or “Lachman*”, not “Lachmans”).
4	If too many records are found, navigate back to the initial search query by clicking “Continue Searching” at the top of the “Search results” page, edit the initial search query by adding another search term to an unused search field, and check ‘Match all search terms (AND)’ is selected at the bottom of the search page.

DiTA has a few useful ancillary features. Search results can be saved and exported by email. The emailed search results can be imported into reference management tools like Endnote. DiTA also allows the user to set up automatic email notification of newly indexed evidence. The DiTA website provides two online tutorials (“Is this study valid?” and “How can I use evidence of diagnostic text accuracy?”) to help users interpret the evidence they find.

**Fig. 2:**
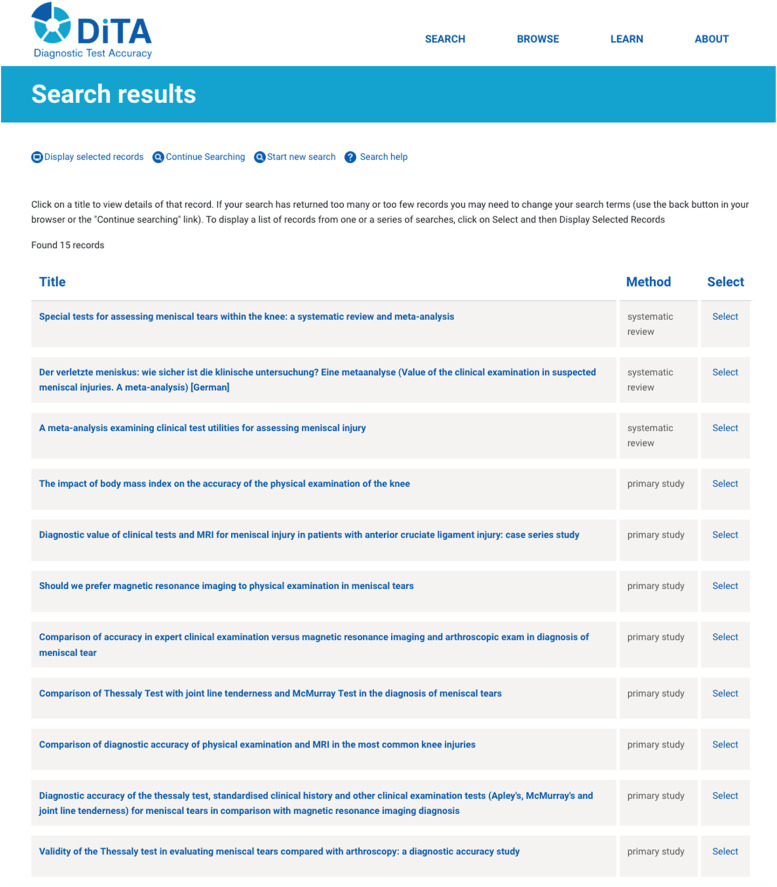
DiTA search results page.

The authors recently conducted a user experience study of the DiTA website and search interface (manuscript under review). That study found participants rated DiTA above average compared to similar web platforms. Participants also reported that they quickly learned to use DiTA. However, participants were commonly observed to use DiTA in a suboptimal way. Examples included the unnecessary use of multiple search fields when fewer would have been more effective, not using the most appropriate search field available (for example, using “Title Only” when using the “Abstract & Title” field was more appropriate), incorrect spelling of search terms, and mis-specification of DiTA-specific search syntax such as wildcards (e.g. *) and Boolean operator functions. To use DiTA most efficiently, users of DiTA should usually enter data into only one or two search fields in an initial search. If only one field is used then it is best to use the “Abstract & Title” field. When using the “Abstract & Title” field, it is best to use two or three simple search terms. The search terms should be separated with spaces – not with Boolean operators (‘AND’ and ‘OR’). Spelling should be checked, especially the use of apostrophes for specific index test names such as ‘Lachman's test’ (‘Lachman's’ retrieves 51 records whereas ‘Lachmans’ retrieves 0 records). If the initial search retrieves too many records, subsequent searches can refine the initial search by using one or more of the unused search fields and checking that ‘AND’ is selected at the bottom of the search page to make the query more specific. Users can navigate back to the initial search query by clicking “Continue Searching” at the top of the “Search results” page (Fig. 2).

On the PEDro database, users can view quality rating scores for each randomized controlled trial indexed on the database. The scores on PEDro are generated using the PEDro scale.^39^ This scale gives a score out of 10 which is intended to represent the methodological quality of randomized controlled trials in physical therapy. When displaying the search results for users, PEDro orders the records for randomized controlled trials from highest to lowest score to help users find the best quality evidence amongst the retrieved studies.^40^, ^41^, ^42^ It would be convenient if the DiTA database could incorporate similar features. Many quality assessment tools have been designed for the purpose of rating the quality of primary studies of diagnostic test accuracy.^43^^,^^44^ Unfortunately, these tools have been shown to be unreliable, which implies that they should not be trusted to provide robust evidence of study quality.^45^ For that reason, DiTA unlike PEDro, does not provide quality rating scores for primary studies, and search results are not returned in order of presumed methodological quality.

As of March 2024, there were 2185 primary studies of diagnostic test accuracy and 277 systematic reviews of primary studies of diagnostic test accuracy indexed on DiTA. The first primary study was published in 1951^46^ and the first systematic review was published in 1995.^47^ The studies have been reported in 19 different languages, with a large majority in English (2315/2462; 94%). Within the first four years of its launch, the DiTA database had been accessed from almost every country in the world. Brazil was by far the largest user of DiTA: 31% of search sessions in that period were conducted from Brazil.

## Conclusion

Diagnostic tests are widely used by physical therapists. It can be difficult to choose which diagnostic tests to apply in a particular situation. The choice of diagnostic test should be informed in part by evidence of test accuracy. Until recently, this evidence was not easy to find. However, the launch of the DiTA database in 2019 has made it easier for physical therapists to find evidence of diagnostic test accuracy. DiTA indexes over 2400 primary studies and systematic reviews of diagnostic test accuracy relevant to physical therapy practice. Access to the database is free. DiTA is used widely across the world, with the largest number of searches being conducted from Brazil.

## Funding

This research did not receive any specific grant from funding agencies in the public, commercial, or not-for-profit sectors. The authors affirm that they have no financial affiliation (including research funding) or involvement with any commercial organization that has a direct financial interest in any matter included in this manuscript, except as disclosed in an attachment and cited in the manuscript.

## Conflicts of interest

The authors declare no conflicts of interest.
